# Triboelectric metamaterial stents: self-powered motility monitoring in post-esophagectomy conduits

**DOI:** 10.1097/MS9.0000000000004905

**Published:** 2026-04-02

**Authors:** Zarqa Yasin, Maryam Nasir, Noor ul Ain Saleem, Abdul Haseeb Hasan

**Affiliations:** a Department of Internal Medicine, Henry Ford Jackson Hospital, MI, USA; b Department of Medicine, Ivane Javakhishvili Tbilisi State University, Tbilisi, Georgia; c Department of Medicine, FMH College of Medicine and Dentistry, Lahore, Pakistan; d Department of Medicine, King Edward Medical University, Lahore, Pakistan; e Department of Research, Oli Health Magazine Organization, Research and Education, Kigali, Rwanda; f Department of Internal Medicine, Sentara Northern Virginia Medical Center, VA, USA

**Keywords:** esophagectomy, leak, monitoring, stent, triboelectric

## Abstract

Anastomotic leaks following esophagectomy remain a major source of morbidity and mortality, yet current postoperative surveillance relies on intermittent imaging and clinical assessment, which may delay recognition. There is a paucity of objective, ongoing gastric conduit function monitoring. We suggest a self-powered stent made of triboelectric metamaterials that produce electrical signals by capturing mechanical energy from peristaltic motion. The conduit’s cyclic deformation generates measurable intraluminal pressure data that is wirelessly delivered for analysis in real time. Motility profiles that deviate from expectations may be early signs of anastomotic compromise. While earlier research validates the accuracy of intraluminal pressure monitoring in the gastrointestinal tract, recent developments in triboelectric nanogenerators and implantable biodegradable devices support the viability of this strategy. A unique approach to ongoing postoperative surveillance is provided by integrating triboelectric energy harvesting with stent design, which may enable earlier leak detection and improve patient outcomes.

*Letter to Editor*,

Anastomotic leaks after esophagectomy are associated with a significantly higher risk of mortality and reduced long-term survival, with meta-analyses demonstrating worse overall survival among patients who develop leaks compared to those who do not[1]. Current postoperative surveillance relies on intermittent imaging and clinical indicators, which have variable diagnostic performance and may not detect anastomotic leaks early after esophagectomy. Because timely recognition is important for patient outcomes, clinical assessment alone can sometimes delay intervention[2].

The key challenge in postoperative esophagectomy care is the absence of continuous, objective monitoring of gastric conduit function. We propose a self-powered gastric conduit stent composed of triboelectric metamaterials that harvest mechanical energy from peristaltic motion to generate electrical signals. Through contact electrification driven by physiological movement, the device would enable real-time, wireless measurement of intraluminal pressure, providing an early indicator of anastomotic compromise before clinical deterioration becomes evident. The proposed system involves the deployment of a flexible triboelectric metamaterial stent within the gastric conduit. Cyclic deformation induced by peristalsis generates electrical output proportional to local mechanical forces. This signal is converted into quantitative intraluminal pressure data and wirelessly transmitted to an external receiver. Temporal pressure patterns can then be analyzed to identify deviations from expected postoperative motility profiles, with alerts triggered by abnormal pressure patterns.

Recent advances support the feasibility of this concept. Triboelectric nanogenerators (TENG) have demonstrated that contact electrification can convert mechanical motion into stable and quantifiable electrical output suitable for biomedical sensing applications[3]. Biodegradable and implantable triboelectric systems have been successfully deployed *in vivo*, demonstrating compatibility with physiological environments and suitability for temporary postoperative use[4]. Furthermore, emerging reports indicate that implantable TENG designs have already been developed with features such as stretchability, bioadhesion, biodegradability, and stimuli responsiveness to adapt to physiological conditions[5]. Finally, ingestible and intraluminal pressure sensors have shown that the gastrointestinal tract supports reliable real-time pressure monitoring[6], establishing a foundation for self-powered conduit surveillance.

Continuous intraluminal pressure monitoring with a self-powered triboelectric stent could allow clinicians to detect abnormal motility or pressure elevations early, enabling timely intervention and reducing reliance on intermittent imaging or subjective assessment.

In conclusion, integrating triboelectric energy harvesting with metamaterial-based stent design offers a novel strategy for postoperative monitoring after esophagectomy. By leveraging peristaltic motion as an intrinsic power source, this approach enables continuous, objective assessment of gastric conduit integrity using existing physiological signals. We encourage studies to evaluate the clinical potential of this device for early detection of anastomotic leaks.

## Data Availability

Data sharing not applicable to this article as no datasets were generated or analyzed during the current study.

## Declaration of generative Al and Al-assisted technologies in the writing process

There is nothing to disclose.

## References

[1] Gujjuri RR Kamarajah SK. Markar SR. Effect of anastomotic leaks on long-term survival after oesophagectomy for oesophageal cancer: systematic review and meta-analysis. Dis Esophagus Off J Int Soc Dis Esophagus 2021;34:doaa085. 10.1093/dote/doaa085 32901259

[2] Moon SW. Kim JJ Cho DG. Early detection of complications: anastomotic leakage. J Thorac Dis 2019;11:S805–S811. 31080662 10.21037/jtd.2018.11.55 PMC6503265

[3] Ranjan N Izadi Z Gaiser P. Contact electrification via redox-active molecules. Angew Chem Int Ed Engl Published online 19 November 2025:e10031. doi:10.1002/anie.202510031 41261844 PMC12759219

[4] Jian G Zhu S Yuan X. Biodegradable triboelectric nanogenerator as a implantable power source for embedded medicine devices. NPG Asia Mater 2024;16:12.

[5] Xiao X Meng X Kwon YH. Emerging triboelectric nanogenerators for in-body implantation. Small Methods Published online 21 October 2025:e01443. doi:10.1002/smtd.202501443 41121587 PMC12893314

[6] Benken A. Gianchandani Y. Passive wireless pressure sensing for gastric manometry. Micromachines 2019;10:868. 31835529 10.3390/mi10120868 PMC6952889

